# New Appliances and Things Medical

**Published:** 1895-06-15

**Authors:** 


### NEW APPLIANCES AND THINGS MEDICAL.

[We shall be glad to receive, at our Office, 428, Strand, London, W.C., from the manufacturers, specimens of all new preparations and appliances which may be brought out from time to time.]

### THE "MOVILLETTE" PINCE-NEZ.

We have been asked by Messrs. J. Raphael and Co., the patentees of the "Movillette" frame for glasses, to reconsider the remarks made about it in our issue of May 11th, remarks which they think did something less than justice to the merits of the invention. A pince-nez frame should keep the major axes of its two lenses on the same horizontal line, should keep the lenses either in a vertical position before the eyes, or inclined forward at the top in any desired degree, should not be liable to fall off, and should not exert any pressure which is irksome to the wearer. There are many noses for which these requirements can be fulfilled by almost any of the common forms of frame; there are many for which they cannot be fulfilled by any of them; and we have seen a great many ingenious frames which had the sole fault of being only fitted for a small number of noses. At the request of Messrs. Raphael, we have tested their power of fitting an exceptionally difficult nose, and have pleasure in saying that they succeeded perfectly; while the projecting spurs in front of the frame, which, as we noticed, come somewhat into the field of vision when the frame is empty, cease to be conspicuous when it is supplied with glasses suited to the wants of the wearer. We may also mention it as an advantage that the frame can be correctly placed in position with one hand. Personally, we do not like any pince-nez as well as spectacles; but that is a question of taste which each wearer must decide for himself.

